# The development of pre-service biology teachers' intention to use digital incremental scaffolds: insights from guided online training

**DOI:** 10.3389/fpsyg.2025.1656795

**Published:** 2025-10-24

**Authors:** Margit Offermann, Rebekka Karbstein, Ricarda Lohrsträter, Nadine Großmann, Jörg Großschedl, Lea Gussen, Svea Isabel Kleinert, Steffen Schaal, Pascal Schaldach, Matthias Wilde

**Affiliations:** ^1^Department of Biology Didactics (Zoology/Humanbiology), Bielefeld University, Bielefeld, Germany; ^2^Institute for Biology, University of Education Ludwigsburg, Ludwigsburg, Germany; ^3^Institute of Biology Education, University of Cologne, Cologne, Germany; ^4^Biology Education Research and Learning Lab (BERLL), University of Duisburg-Essen, Essen, Germany

**Keywords:** behavioral intention, pre-service teachers, professional development, teacher training, TPACK, scaffolds

## Abstract

**Introduction:**

In an increasingly digital educational landscape, it is essential that teachers not only possess subject-specific knowledge but are also able to integrate digital tools in their teaching. This study investigates the impact of an asynchronous digital teacher training course on pre-service biology teachers' behavioral intentions regarding the implementation of digital incremental scaffolds. Based on the Theory of Planned Behavior (TPB) and the Technological Pedagogical and Content Knowledge (TPACK) framework, this study explores psychological and pedagogical factors that predict such behavioral changes.

**Methods:**

As an intervention, an iMooX-hosted training comprised eight modules, divided into an introductory unit, a basic module and three advanced modules to support the development and implementation of digital incremental scaffolds in biology lessons. A total of 100 German pre-service biology teachers (*M* = 23.4 years, *SD* = 3.5, 86% female) participated in this quasi-experimental pre-post intervention study as part of their university curriculum. They completed standardized self-reporting questionnaires before and after engaging in the 3-h asynchronous digital teacher training course. The test instruments assessed constructs from the TPB and TPACK frameworks. Paired *t*-tests and structural equation modeling were used for data analysis.

**Results:**

There were significant increases across all sub-dimensions of the TPB, with the highest effective sizes in attitude (*d* = 0.86) and self-efficacy (*d* = 1.33); as well as behavioral intention itself (*d* = 0.80) and TPACK (*d* = 0.70). A regression analysis showed that attitude (β = 0.66, *p* < 0.001) and subjective norm (β = 0.18, *p* < 0.01) significantly predicted behavioral intention, while TPACK (β = 0.35, *p* < 0.001) and attitude (β = 0.57, *p* < 0.001) significantly predicted self-efficacy.

**Discussion:**

These findings support the relevance of internal beliefs and social expectations in driving pre-service teachers' intention to integrate digital tools. The results also emphasize the importance of pedagogical-technological knowledge in shaping teachers' confidence in implementing such digital tools. The study's implications for designing practical informed digital training formats in teacher education are discussed.

## 1 Introduction

In the ongoing digital transformation in education, digital tools and platforms become increasingly integral to classroom practice. It is essential to consider how teachers—both those in training and those already in the profession—are prepared for such evolving demands. In particular, the growing expectation for digitally supported and differentiated instruction calls for teacher education programs (Hmelo-Silver et al., 2007; Hörmann et al., 2024). Digital learning platforms offer promising opportunities for flexible learning environments (OECD, 2023). Compared to traditional in-person workshops, digital training formats might improve work-life balance and increase engagement with educational technologies, to enhance teaching practices and student learning outcomes (Hörmann et al., 2024). However, it is essential to recognize that pre-service and in-service teacher education serve distinct yet complementary purposes. While in-service training typically builds on existing classroom experience and professional competence, pre-service programs focus on establishing the foundational knowledge, beliefs, and attitudes necessary for future professional development (Lipowsky and Rzejak, 2015). One widely recognized framework for digital teaching competence is Technological Pedagogical and Content Knowledge (TPACK). TPACK captures the interplay between content knowledge, pedagogical knowledge, and technological tools. Originally conceptualized as a form of integrated knowledge (Mishra and Koehler, 2006), recent work has also emphasized its value as a practical application that informs instructional design (Willermark, 2018). In the context of biology education, TPACK plays a particularly crucial role (Miller and Yoon, 2023). Teachers must not only master the subject matter but also learn how to use digital technologies to support diverse learners. Moreover, research increasingly emphasizes that, beyond competence, motivational constructs such as self-efficacy beliefs and behavioral intentions are crucial for translating knowledge into actual classroom practice (Bertram et al., 2023; Mkhomi et al., 2025).

Against this backdrop, the present study investigated the impact of a digital training course designed for both pre-service and in-service biology teachers to examine whether this intervention could strengthen pre-service teachers' behavioral intention to implement digital incremental scaffolds in their classrooms (Fleischer et al., 2023). Grounded in the Theory of Planned Behavior (Ajzen, 1991), the present study explores whether its subdimensions (i.e., self-efficacy; Bandura, 1997) or TPACK itself can serve as predictors of this intention. To address these interrelated questions, an asynchronous online course was developed and hosted on the Moodle-based iMooX platform. The course was designed to offer structured and engaging digital learning opportunities through a combination of theoretical content, practical examples, reflection prompts, and self-assessment tools.

## 2 Theoretical background

### 2.1 The TPACK framework in the context of digital education

**Figure 1:**
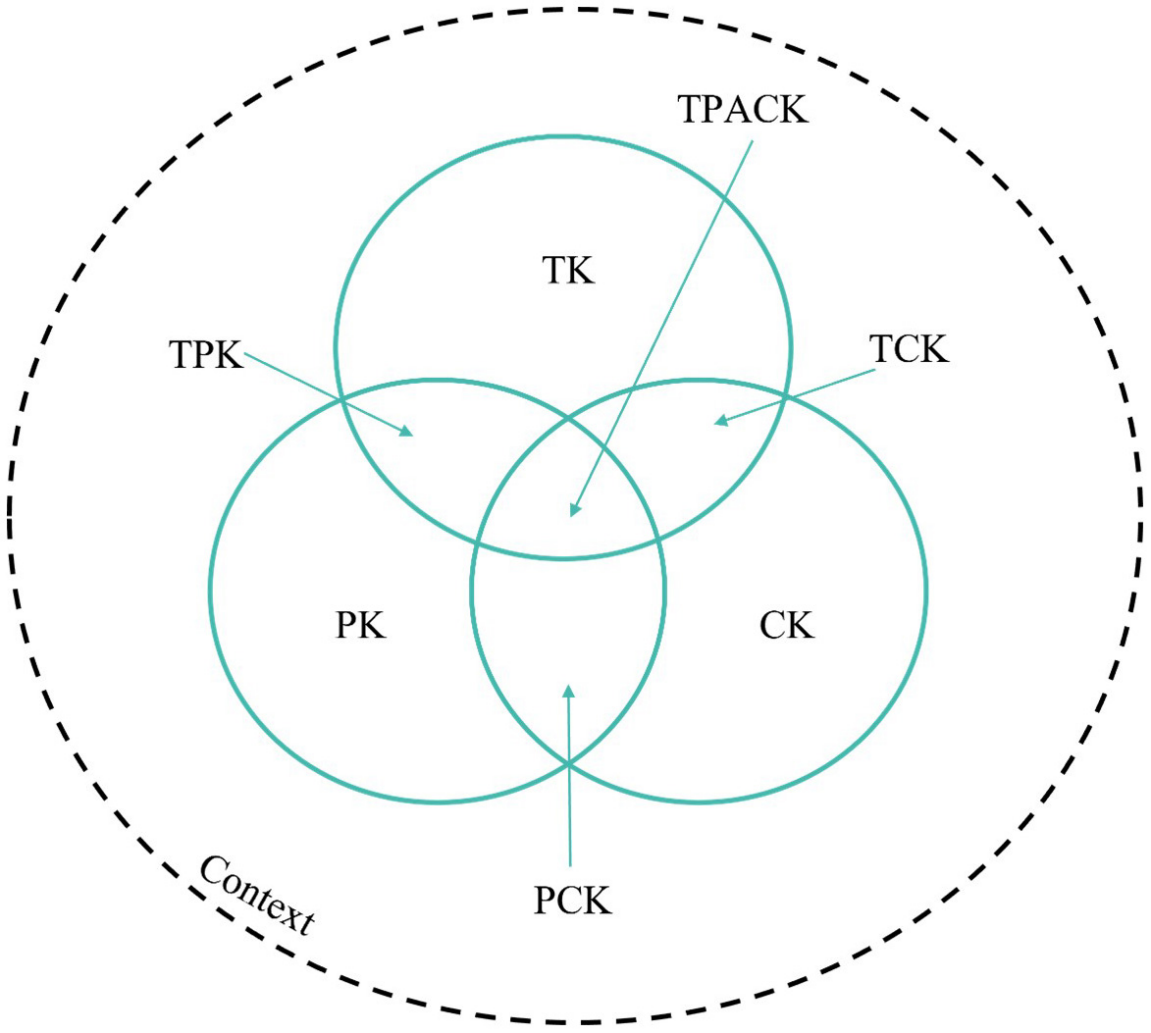
The TPACK framework and its knowledge dimensions (after Koehler et al., 2014).

The Technological Pedagogical and Content Knowledge (TPACK) framework (see Figure 1; Koehler et al., 2017) posits that teachers need to master three primary domains of knowledge—technological, pedagogical, and content-specific—to integrate digital tools into their teaching effectively (Huwer et al., 2019). TPACK thus serves as a valuable model for diagnosing and developing teachers' ability for digital integration in complex classroom settings.

Content Knowledge (CK) refers to teachers' expertise in their academic discipline (Baumert and Kunter, 2006; Shulman, 1986). In the context of biology education, this encompasses knowledge of key topics, including genetics, cell biology, ecology, and evolution (Gimbel et al., 2021). A solid grasp of these areas is essential for accurate and meaningful instruction (Mishra and Koehler, 2006). Pedagogical Knowledge (PK) encompasses general knowledge of teaching and learning processes, including classroom management, student motivation, and assessment techniques (Park and Oliver, 2008). For example, inquiry-based learning and differentiated instruction are expressions of PK (Shulman, 1986). Technological Knowledge (TK) involves familiarity with digital tools and their functions, independent of specific pedagogical applications (Mishra and Koehler, 2006). This includes knowing how to operate a digital microscope or setting up an interactive whiteboard. Pedagogical Content Knowledge (PCK) refers to the ability to transform the subject matter into teachable content by using suitable didactic methods. For example, a biology teacher may use models or analogies to explain concepts such as photosynthesis or genetic inheritance (Shulman, 1986). Technological Content Knowledge (TCK) denotes an understanding of how digital tools can enhance the teaching of specific subject content. An example would be using a virtual simulation to model population dynamics in an ecosystem or employing 3D animations to explain molecular structures (Mishra and Koehler, 2006). Technological Pedagogical Knowledge (TPK) refers to the ability to use technology to support broader educational strategies. A teacher with strong TPK might, for example, employ polling tools such as Mentimeter to support formative assessment (Koehler et al., 2017). TPACK ultimately represents the integrative understanding that enables teachers to align all three domains—technology, pedagogy, and content—in a cohesive and context-sensitive manner (Thyssen et al., 2023). Teachers with high TPACK can design digital learning environments that are not only technically functional but also pedagogically meaningful and aligned with curricular goals (Hew et al., 2019). Figure 1 illustrates the interconnections among these seven knowledge domains.

As mentioned, TPACK is particularly important in STEM education to visualize abstract or complex phenomena (Roth et al., 2023). Importantly, teachers must not only be technically proficient but also able to critically assess when and how to implement digital tools effectively and in pedagogically sound ways [KMK (Kultusministerkonferenz), 2021; Punie and Redecker, 2017; Seufert et al., 2021; Smetana and Bell, 2012]. For example, using simulation software to model ecological interactions or digital microscopes to explore cell structures, as well as an interactive phylogenetic tree tool to enhance students' understanding of evolutionary relationships are typical applications of digital tools in biology instruction (Bwalya et al., 2023, 2024). Bwalya et al. (2024) also report that pre-service biology teachers' engagement in reflection upon technology usage and collaboratively designing lessons could improve TPACK domains. While TPACK focuses on integrated learning, the Theory of Planned Behavior offers a complementary perspective on motivational factors that shape the intention to use such knowledge in practice.

In the present study, TPACK is not only understood as a theoretical framework of digital teaching competence, but also as a central learning outcome targeted by the digital training course developed for pre-service biology teachers. Accordingly, we examine whether participation in the course leads to measurable gains in TPACK (RQ1) and whether TPACK interacts with motivational beliefs as a predictor of self-efficacy (RQ3).

### 2.2 The theory of planned behavior in the context of digital education

The Theory of Planned Behavior (TPB) is a well-established psychological model that explains and predicts human behavior. The theory provides a framework for analyzing why teachers choose to adopt (or avoid) digital technologies and under what conditions they are willing to do so (Ajzen, 1991; Chen and Slade, 2025).

**Figure 2:**
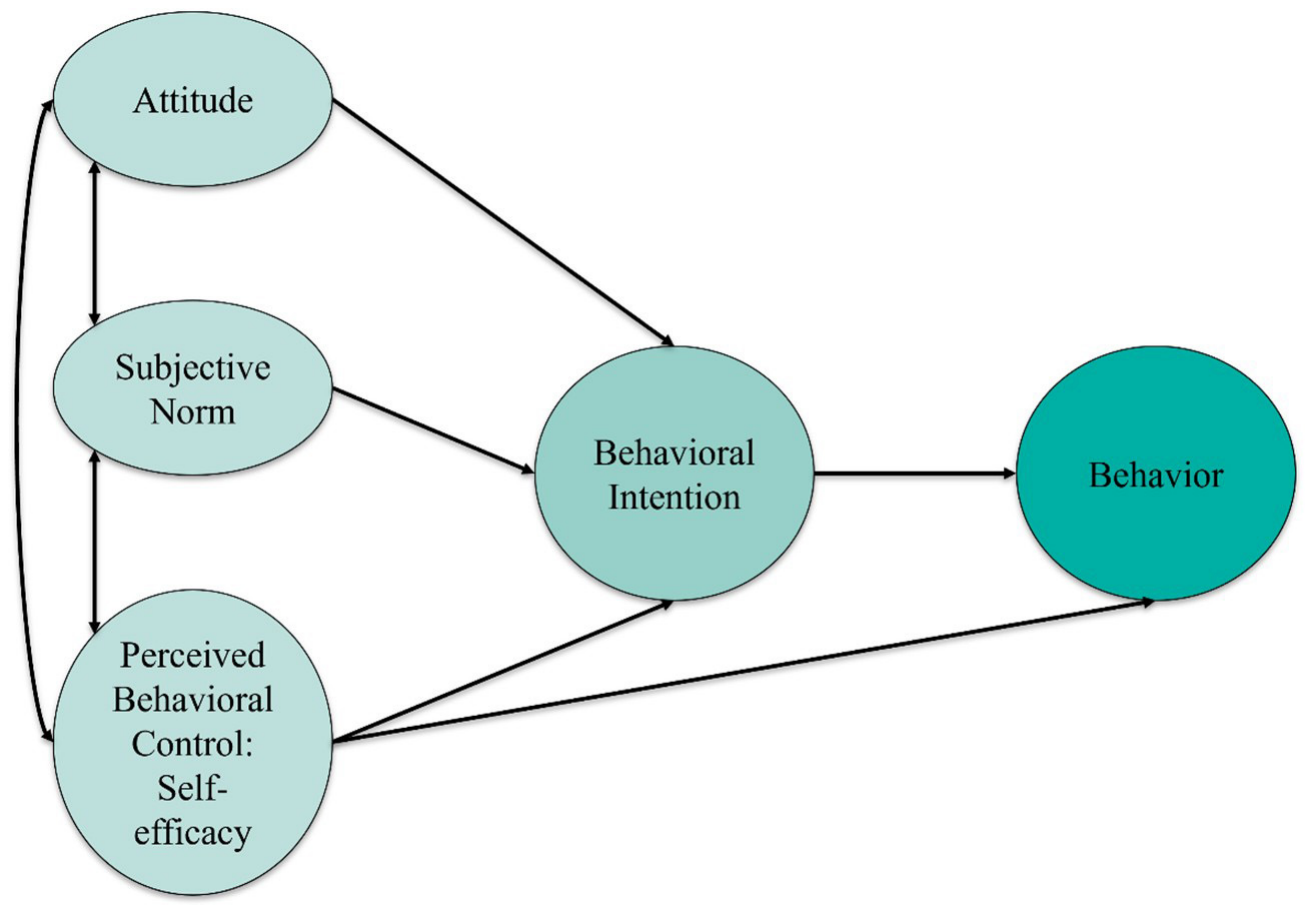
A conceptual model of the Theory of Planned Behavior (Ajzen, 1991), illustrating the relationships between attitude, subjective norm, perceived behavioral control (here interpreted as self-efficacy), behavioral intention, and behavior (adapted based on Leiss et al., 2025). ^*^p < 0.05; ^***^p < 0.001.

In recent years, the theory has been applied in teacher education research to explain behavioral patterns related to the use of instructional approaches, such as digital tools (Hou et al., 2022; Sadaf and Johnson, 2017). According to TPB, the formation of an intention to perform a behavior—as well as the behavior itself—is shaped by three central psychological components: attitude, subjective norm, and perceived behavioral control (see Figure 2; Ajzen, 1991). These components collectively explain how individuals form behavioral intentions and how these intentions translate into specific actions.

#### 2.2.1 (Pre-service) teachers' attitude, subjective norm, and self-efficacy toward digital education

Attitude toward a behavior is one of the three primary predictors of behavioral intention, which in turn influences actual behavior. Attitude refers to an individual's overall evaluation, which is favorable or unfavorable, toward performing a specific behavior (Ajzen, 1991). For example, if teachers believe that using digital technology enhances learning outcomes and student engagement, they are more likely to intend to integrate such tools into their lessons.

For pre-service teachers, attitude plays a pivotal role in shaping their intention to utilize educational tools such as technology-enabled learning Hou et al., (2022); Scherer et al., (2019). A positive attitude is often driven by underlying beliefs about the expected outcomes of technology use, such as improved student engagement or instructional effectiveness. (Hou et al. 2022) show that pre-service teachers who perceive digital tools as beneficial are significantly more likely to express a stronger intention to integrate them into their future practice. Further research also highlights that attitude mediates the relationship between digital competence and behavioral intention, suggesting that merely acquiring technical skill is insufficient unless accompanied by positive emotional and cognitive dispositions toward technology use (Alieto et al., 2024; Galindo-Domínguez et al., 2024). Therefore, fostering constructive attitudes during teacher training is crucial for developing the motivation and commitment necessary to implement digital innovations in the classroom effectively. Positive expectations—for example, the belief that educational technology can enhance student engagement and learning—have been shown to significantly bolster pre-service teachers' intentions to adopt digital methods Nair and Karan, (2024); Önal, 2024). (Nair and Karan 2024) found that without a favorable disposition toward technology, mere competence failed to translate into the behavioral intention needed for classroom implementation.

Subjective norm refers to the perceived social pressure to perform or avoid a given behavior. In the school context, this can mean that teachers are more inclined to use digital media if they perceive expectations from colleagues, school administrators, or parents to do so. Perceived behavioral control reflects the perceived ease or difficulty of performing the behavior, resembling the concept of self-efficacy. The concept encompasses teachers' beliefs regarding whether they possess the necessary competencies and resources—such as technical infrastructure or professional support—to effectively utilize digital tools in their teaching. These three variables influence behavioral intention (see Figure 2), a factor that is considered the most immediate predictor of actual behavior. The stronger the intention, the more likely the behavior will follow, particularly when the perceived behavioral control is high (Ajzen, 1991; Chen and Slade, 2025).

Self-efficacy in teaching refers to a teacher's confidence in their ability to influence student engagement and learning, even in challenging situations (Lazarides and Warner, 2020; Thommen et al., 2022). The concept represents a context-specific judgment of how well a teacher is able to successfully carry out the instructional tasks required to achieve desired educational outcomes. High self-efficacy is associated with greater openness to new teaching methods, more effective planning and organization, increased perseverance in the face of challenges, and a greater use of innovative and student-centered teaching strategies (Lazarides and Warner, 2020). According to Bandura's social cognitive theory, self-efficacy refers to an individual's belief in their ability to achieve intended outcomes through purposeful action (Bandura, 1997). Teachers with high self-efficacy are more likely to adopt technological tools, adapt to changing demands, and respond to the diverse needs of their students in digital learning environments (Scherer et al., 2018). They tend to perceive challenges as opportunities for professional growth; this can positively affect the classroom climate, student motivation, and learning outcomes (Tschannen-Moran and Hoy, 2007). Moreover, such teachers demonstrate greater resilience in the face of setbacks and are better equipped to implement differentiated instruction, manage classroom behavior, and maintain high expectations for all learners. Empirical studies suggest that self-efficacy is not a stable personality trait but can be developed through targeted interventions (Bandura, 1997; Klassen and Tze, 2014). These include professional learning experiences, mastery of subject matter, observing competent colleagues (modeling), and receiving constructive (peer) feedback. Structural support measures such as collegial collaboration, mentoring programs, and targeted professional development can also enhance the development of self-efficacy. In a digital teaching context, hands-on experience with technology, continuous reflection, and pedagogical guidance are particularly relevant (Thommen et al., 2022). Therefore, educational institutions could significantly contribute to the professionalization of teachers.

In the present study, TPB serves as the motivational framework to explain pre-service biology teachers' intention to implement digital incremental scaffolds. Specifically, we examine how attitude, subjective norm, and self-efficacy (as part of perceived behavioral control) predict behavioral intention (RQ2). Furthermore, we analyze how these motivational factors interact with technological-pedagogical competence (TPACK) in shaping self-efficacy and intention (RQ3).

### 2.3 Professional development through digital teacher training

The professional development of teachers encompasses structured activities and learning experiences designed to enhance competence, knowledge, and pedagogical expertise. This way, teaching practices and student outcomes should improve Mullis et al., (2016). These activities may take form of workshops, training courses or seminars. They include experiences that expand teachers' instructional capacities in ways that positively impact student learning (Darling-Hammond et al., 2017; Makhmetova et al., 2025). Importantly, the professional development needs of pre-service and in-service teachers differ considerably due to variation in professional identity, classroom experience, and developmental priorities (Makhmetova et al., 2025). Pre-service teachers—typically at the start of their professional trajectory—focus on foundational experiences that help them build confidence and self-efficacy in classroom settings. According to Dewhurst and McMurtry (2006), placement experiences that foster a sense of belonging and provide continuous feedback are particularly beneficial in this early stage (Mkhomi et al., 2025). In contrast, in-service teachers usually approach professional development from a perspective of refinement and application. Their learning tends to be more self-directed and grounded in classroom reality (Darling-Hammond et al., 2017). Research by (Evişen 2021) has shown that experienced teachers value professional development formats that are practical, collaborative, and contextually relevant. Whereas pre-service teachers often rely on structured input and feedback to monitor progress, in-service teachers prioritize peer exchange, professional dialogue, and direct applicability to everyday classroom practice (Ansyari et al., 2022; Cleaver et al., 2020; Evişen, 2021; Darling-Hammond et al., 2017). Attitudes toward educational technologies also vary across these groups. Pre-service teachers are generally more open to digital settings and tend to perceive them as engaging and efficient (Paetsch and Drechsel, 2021). Their greater familiarity with digital tools enables them to easily integrate learning management systems and online communities into their professional learning. In contrast, in-service teachers are often more critical and pragmatic in their use of technology (Abid et al., 2022). Feedback for in-service teachers is most effective when it emerges from collaborative engagement rather than from externally imposed assessments (see Dinata et al., (2022). Acknowledging these distinct developmental needs allows for the design of professional development formats that effectively support teacher learning across different enables the design of professional development formats that effectively support teacher learning across various career stages and contexts.

#### 2.3.1 Professionalization in teaching—requirements, formats, and effectiveness of continuing professional development in the digital age

Continuous professional development is a central component of professional teaching practice. Training programs that specifically aim to enhance profession-related competencies enable teachers to respond flexibly to educational challenges (KMK (Kultusministerkonferenz), 2021; Lipowsky, 2010; Lipowsky and Rzejak, 2021; OECD, 2019). Both the Standing Conference of the Ministers of Education and Cultural Affairs (KMK (Kultusministerkonferenz), 2021) and the OECD (2019), based on findings from the TALIS study, emphasize that professional development should support the advancement of subject-specific, pedagogical, and organizational competence. This way, teachers should be prepared for current demands and challenges such as heterogeneity, inclusion, and digitalization. Digital competencies are becoming increasingly important in education (Punie and Redecker, 2017). Teachers must not only master the technical use of digital tools such as learning platforms or feedback systems but also implement such tools to foster individual support, differentiation, and self-regulated learning (Koehler and Mishra, 2009; OECD, 2021). The integration of these competencies requires a combination of content knowledge, pedagogy, and technology, as outlined in the TPACK model (Koehler and Mishra, 2009).

The effectiveness of professional development measures depends largely on the alignment between training content and the individual development needs of teachers (Kramer et al., 2024; Lipowsky and Rzejak, 2021; Rzejak et al., 2024). At the same time, barriers such as limited time, insufficient technical infrastructure or lack of quality standards, frequently hinder the implementation and impact of training measures [Ständige Wissenschaftliche Kommission der Kultusministerkonferenz (SWK), 2023; Wanitschek et al., 2020]. A key factor in the effectiveness of training is the alignment between the training content and the individual professional development needs of teachers Kramer et al., (2024); Lipowsky and Rzejak, (2021); Rzejak et al., (2024). Effective programs are characterized by clear objectives, content relevance, a strong connection between theory and practice, collaborative learning, and opportunities for feedback and reflection (Desimone, 2009; Lipowsky and Rzejak, 2021; Rzejak et al., 2024). Professional development opportunities that address hands-on application elements contribute to teacher professionalization and the overall improvement of teaching quality in schools (Castañeda and Selwyn, 2018; Runge et al., 2024).

#### 2.3.2 Professionalization goal: implementation of digital incremental scaffolds

Incremental scaffolds can be implemented in both analog and digital forms. By combining the principles of digital differentiation with those of incremental scaffolding, (pre-service) teachers can offer flexible, adaptive support that addresses the diverse learning needs of students in inclusive classroom settings. To reduce the risk of exclusion, (Böttinger and Schulz 2023) emphasize the importance of linking digitization and inclusion. Digital technologies have the potential to create more equitable learning environments by identifying barriers and offering alternative pathways to understanding Abels and Stinken Rösner, (2022). For example, read-aloud functions, subtitling, and visual aids can support learners with varying degrees of language proficiency or cognitive needs (Abels and Stinken Rösner, 2022). In the context of digital incremental scaffolds, such features can be used to adapt content based on individual learners' prior knowledge and experience. Glossaries, explanatory videos, and interactive prompts offer multiple means of access to complex subject matter (Stinken-Rösner et al., 2023). (Greitemann et al. 2021) found that low-performing students, in particular, benefit from these forms of digital support, both in terms of subject-specific knowledge and confidence. Incremental scaffolds are particularly effective for complex tasks that require step-by-step support and structured guidance (Hänze et al., 2010). Such tasks help learners by breaking down complex tasks into manageable tasks and offering graduated prompts or solution strategies. These scaffolds are flexible in design and can be used in individual, pair, or group work. One significant advantage is that they preserve the cognitive complexity of a task while adapting support levels to learners' performance and needs. Students can self-select the level and type of support they receive, from thought-provoking cues to partial solutions (Hänze et al., 2010). In biology education, incremental scaffolds are commonly used to support structured learning processes such as experimental design or hypothesis development and testing (Stäudel et al., 2007). However, despite the potential of this approach, students often struggle with acquiring fundamental scientific competence. Challenges such as formulating testable hypotheses or constructing valid experimental procedures persist in biology classrooms (Fernández et al., 2022), increasing cognitive load (Toh and Tasir, 2024). This highlights the need for adaptive, technology-enhanced strategies that support cognitive engagement (Nurdin et al., 2025). Empirical findings have demonstrated that incremental scaffolds foster scientific reasoning (Arnold et al., 2017) and improve both conceptual understanding (Mustafa et al., 2021) and procedural competence (Stiller and Wilde, 2021). Furthermore, studies have shown positive effects on student motivation, interest, and self-regulation Hänze et al., (2010); Großmann and Wilde, (2019); Kleinert et al., (2022). These motivational gains are particularly important in the context of pre-service teacher education, where personal engagement with digital tools is key to shaping future classroom practice. Building on these theoretical insights, the following section describes the design and structure of a digital training framework developed to promote pre-service biology teachers' intention to use digital incremental scaffolds.

## 3 Research questions

The integration of digital tools in science education has emerged as a pivotal component of contemporary teaching, particularly in the context of supporting students through digital scaffolding strategies. Despite the growing recognition of the benefits of digital incremental scaffolds, there remains a need to understand how training interventions can shape future teachers' behavioral intentions.

Self-assessments in pre-post designs have proven to be a practical and meaningful approach in studies of educational interventions, particularly for illustrating changes in competence and attitudes (König et al., 2025). By focusing on biology education, this study seeks also to provide empirical insights into how teacher preparation programs can be optimized to support the integration of digital scaffolds in future teaching practices.

Building on this approach, we investigated the impact of a digital training course on the behavioral intention of pre-service biology teachers to implement digital scaffolding strategies in the classroom after participating in the training. A secondary goal was to identify the psychological and pedagogical factors that influenced this outcome and how these factors are interrelated, drawing on the Technological Pedagogical and Content Knowledge (TPACK) framework and the Theory of Planned Behavior (TPB; Ajzen, 1991). Our study addresses the following research questions:

RQ1: Does participation in a digital training course lead to changes in pre-service biology teachers' TPACK and behavioral intention regarding the use of digital scaffolding strategies in the classroom?
RQ2: To what extent do TPACK and the TPB sub-components (attitude, subjective norm, and self-efficacy) contribute to pre-service teachers' behavioral intention to implement digital scaffolding strategies?
RQ3: To what extent do TPACK and the TPB sub-components (attitude and subjective norm) contribute to teachers' self-efficacy concerning the use of digital scaffolding strategies?

## 4 Methods

### 4.1 Sample

A total of 287 pre-service teachers took part in the digital training as one of the mandatory curricular courses. However, participation in the accompanying scientific surveys (pre- and post-test) was voluntary, resulting in a final sample of *N* = 100 students for the analysis. This substantial reduction in sample size may reflect the non-mandatory nature of participation and competing demands on students' time. It also introduces the possibility of self-selection bias. On one hand, some students may have been more interested in completing the questionnaire or might have had more time at the end of the seminar. Another bias may have occurred by students completing the questionnaire since they were more motivated or interested in digital tools. Therefore, such students may have been more likely to complete the questionnaires. Consequently, the findings should be interpreted with caution, as the sample may not fully represent the entire cohort of participants. The data were collected using a blind design with pseudonyms in compliance with data protection regulations. Among the participants, 86% identified as female, 13% as male, and 1% as diverse. For educational institution, 72% of the students were enrolled in the University of Cologne, 25% in Bielefeld University, and 3% in the Ludwigsburg University of Education. The average age was *M* = 23.4 years (*SD* = 3.5). At the time of the survey, 39% of the subjects were studying for a Bachelor's degree, and 61% for a Master's degree. Approximately one in five (20.9%) of the participants were already familiar with the concept of digital incremental scaffolds before the course.

### 4.2 Research ethics

Participation in the scientific surveys (pre- and post-test) was entirely voluntary. Students were informed that their responses would be treated confidentially and used exclusively for research purposes. They were assured that their participation or non-participation would have no effect on their course results or academic standing, and that they could withdraw at any time without negative consequences. Prior to data collection, all participants provided informed consent by agreeing with the privacy policy. All research involving participants was reviewed and approved by the Ethics Committee of Bielefeld University.

### 4.3 Study design

**Figure 3:**
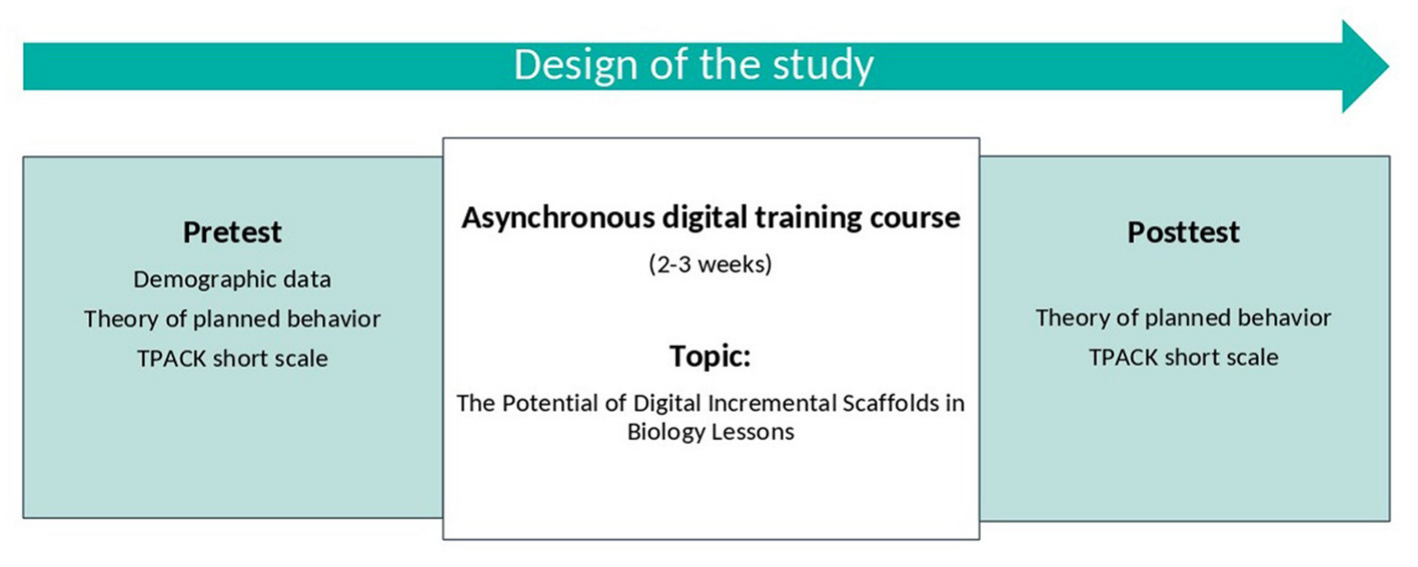
The design of the quantitative pre-post intervention study comprised a pretest, an asynchronous digital training course, and a posttest.

The present study employed a single-group quasi-experimental pre-post design without a control group. This choice was motivated by the institutional context: the training was offered as a regular course unit, that all enrolled students participated and no separate control group could be established. As the training was part of the curriculum, withholding it from a subgroup would have raised organizational and ethical concerns. The design thus allowed us to evaluate the feasibility and short-term effects of the training under realistic teaching conditions, while acknowledging its methodological limitations. The sample comprised pre-service biology teachers participating in a university didactic training course on the integration of digital incremental scaffolds in biology lessons. The data were collected at two measurement points (T1 = before the training; T2 = after completion of the training). In a final sample of *N* = 100, the participants took part in the pretest, posttest, and intervention (see Figure 3). Additional demographic data was collected as part of the pretest in a previous course unit. In the intervention, the participants completed a digital training course on the topic of “The potential of digital incremental scaffolds in biology education” within 2–3 weeks. The posttest was integrated into the final training module. Both surveys were conducted using the online survey platform LimeSurvey (completion time: *M* = 9.70 min, *SD* = 4.10).

To verify the completion of the asynchronous digital training course, pre-service teachers submitted their certificates detailing the lessons they had attended. This was mandatory for completing the base module (lessons 2–5) in full and at least one advanced module (lessons 6–8). A self-assessment was required to be passed for each lesson to verify knowledge transfer. As there were only 2–3 weeks between surveys to complete the training course, it is highly likely that significant increases observed in different constructs were the result of participating in the asynchronous digital training course.

### 4.4 Description of the professional development course

**Figure 4:**
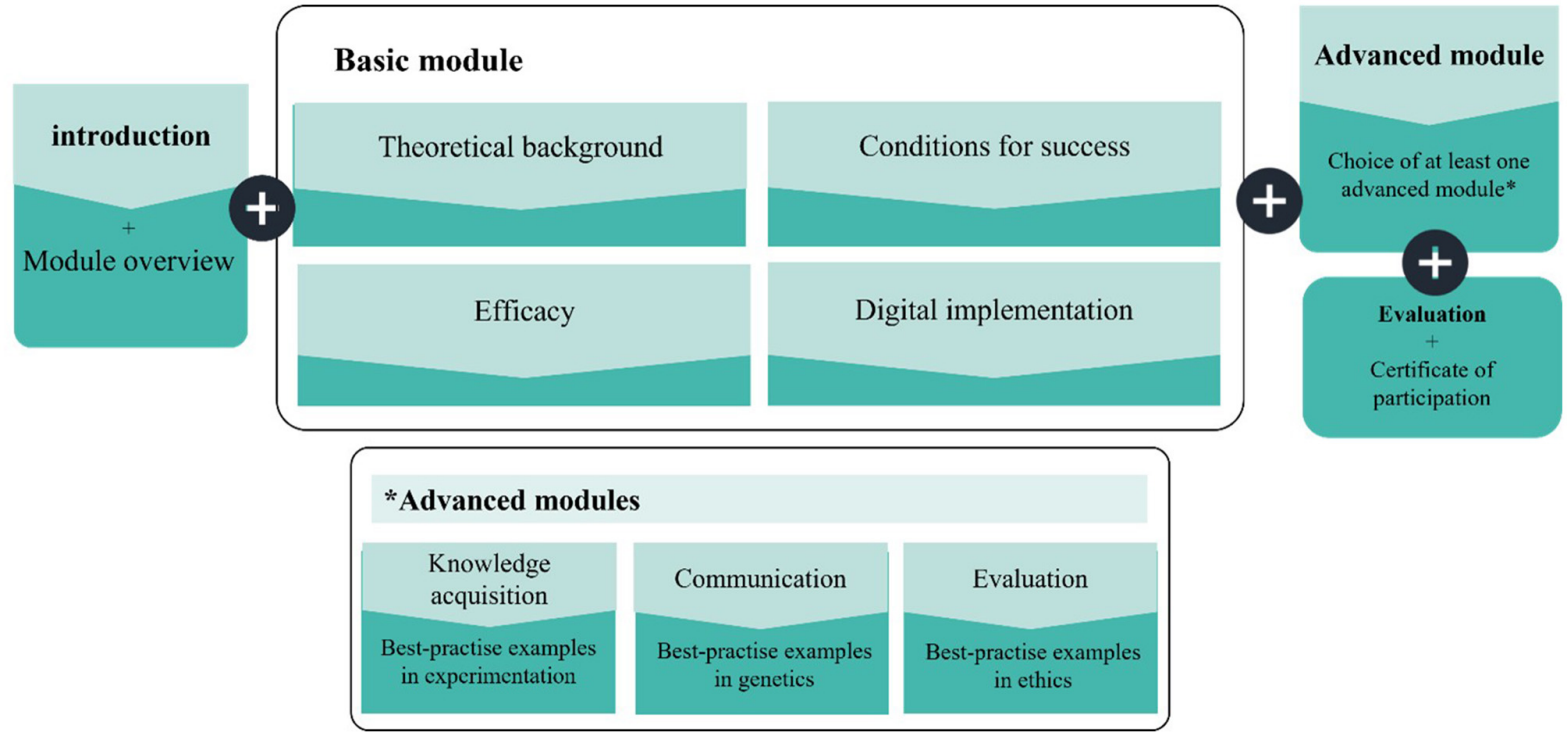
The structure of the digital training course, “The Potential of Digital Scaffolds in Biology Lessons,” including an introduction, a basic module, advanced modules, evaluation, and a certificate.

The effective use of digital incremental scaffolds requires competency in two key aspects: understanding the concept of incremental scaffolding and knowing how to implement it digitally from both technical and pedagogical perspectives. These principles guided the design of the training course described in this study (see Figure 4). The course was aligned with the ten core features of effective professional development identified by Lipowsky and Rzejak (2021). Particular focus was placed on the pedagogical value of incremental scaffolds for promoting self-regulated and meta-cognitive learning. To support practical implementation, the course enabled participants to design scientifically sound materials tailored to the diverse needs of learners.

The digital training course (see Figure 4) introduced the theoretical foundations of incremental scaffolding and demonstrated its practical relevance through authentic examples from biology instruction. Given the complexity of biology teaching—combining abstract content and diverse student needs—the course highlighted the connections between scientific content and differentiated instruction. To facilitate classroom transfer, the course design was guided by the Theory of Planned Behavior (Ajzen, 1991), with the goal of strengthening pre-service teachers' intention to use digital incremental scaffolds. Attitudes toward the use of digital scaffolds were shaped through instructional scenarios, teacher testimonials, and hands-on activities. The participants created their own scaffolds to internalize the value of the approach and reduce reservations. Subjective norm was addressed through asynchronous forums and optional workshops that fostered peer exchange and reinforced social expectations. To enhance the degree of perceived behavioral control, the course offered screencasts, templates, and structured feedback to boost both confidence and competence.

The course was hosted on the Moodle-based platform iMooX and consisted of eight structured lessons in three sections: an introductory unit, a comprehensive basic module (lessons 2–5), and three advanced modules (lessons 6–8). The basic module covered student heterogeneity, didactic principles of scaffolding, the conditions for implementation, and strategies for inclusive digital learning delivered via educational videos, interactive tasks, and biology-specific examples. The basic module concluded with an overview of digital tools, such as QR-based scaffolds. The advanced modules focused on the core scientific competencies in the German biology curricula: knowledge acquisition, communication, and evaluation. Each module included teaching scenarios, instructional videos, scaffold design tasks, and opportunities for feedback. Supplementary readings and quizzes supported reflection and progress monitoring. Overall, the training emphasized practical application, individualized support, and digital literacy, providing a protected space for participants to design, test, and refine scaffold-based teaching materials.

### 4.5 Test instruments

To examine pre-service biology teachers' behavioral dispositions toward the implementation of digital incremental scaffolds, an adapted questionnaire was developed based on several published Theory of Planned Behavior (TPB) scales Aptyka and Großschedl, (2022); Francis et al., (2004); Leiss et al., (2025); Sadaf et al., (2012). The adapted questionnaire has been tested and validated in the context of gamification in biology education by Leiss et al. (2025). We measured four out of the five standard TPB constructs: attitude, subjective norm, perceived behavioral control self-efficacy, and behavioral intention. The construct perceived behavioral control controllability was excluded due to the fixed framework conditions for the participants. All items were adapted to specifically address the use of digital incremental scaffolds in (future) biology lessons.

The final TPB-based instrument comprised 19 items (see Table 1), each rated on a five-point ratingscale ranging from 0 (not at all true) to 4 (completely true). For attitude, four items from Sadaf et al. (2012) were reworded to include the topic of digital incremental scaffolds. In addition, two items were added based on Francis et al. (2004): “The use of digital incremental scaffolds would be/is misleading” and “I think the use of digital incremental scaffolds would be/is effective.” For subjective norm, three items from Sadaf et al. (2012) were similarly adapted, and one item was added based on (Aptyka and Großschedl 2022): “People whose opinion I value will recommend that I use digital incremental scaffolds.” For perceived behavioral control and self-efficacy, three items were adapted from Sadaf et al. (2012), and one item was added: “I can find a solution for every problem when using digital incremental scaffolds in my lessons” Francis et al., (2004); Leiss et al., (2025). For behavioral intention, three items from Sadaf et al. (2012) were modified to include digital incremental scaffolds, and two additional items were added from Francis et al. (2004): “I would like to use digital incremental scaffolds in my lessons in order to do justice to the growing heterogeneity” and “I plan to use digital incremental scaffolds in my lessons to make my teaching more varied.”

**Table 1:** Summary and reliability of the adapted TPB (Francis et al., 2004; Sadaf et al., 2012) scales.

**Scale**	**Number of items**	**Example items**	**Cronbach's** ***α***	
			**Pre-test**	**Post-test**
Subjective norm	4	My superiors (e.g., seminar leader, head teacher) will expect me to use digital incremental scaffolds in my lessons.	0.72	0.74
Perceived behavioral control self-efficacy	4	I could easily create digital incremental scaffolds on my own.	0.76	0.76
Attitude	6	I have the feeling that digital incremental scaffolds are easy to use.	0.75	0.77
Behavioral intention	5	I plan to use digital incremental scaffolds in my future teaching activities.	0.86	0.88

A summary of the TPB test instrument and the corresponding reliability coefficients is presented in Table 1. According to Nunnally and Bernstein (1994), values of Cronbach's alpha (α) ≥0.80 indicate good reliability, while values ≥0.70 are acceptable, and values < 0.69 are considered questionable to poor; values < 0.50 are typically unacceptable. The adapted TPB scales exhibited Cronbach's α values ranging from 0.72 to 0.88, indicating acceptable to good internal consistency.

A short instrument developed by Brändle et al. (in preparation), (in revision) based on (Zinn et al. 2022) was employed to assess participants' perceived technological pedagogical content knowledge (TPACK). The scale consisted of three items per subscale, also rated on a five-point scale with the same response format. All TPACK subscales exhibited acceptable to good reliability (see Table 2). The TPACK scale has been tested and validated by Brändle et al. (in preparation), though item-formulation has been adapted to fit biology education.

**Table 2:** Summary statistics and reliability of the adapted TPACK scales (Brändle et al., in preparation, adapted after Zinn et al., 2022).

**Scale**	**Number of items**	**Example items**	**Cronbach's** ***α***	
			**Pre-test**	**Post-test**
TK	3	I find it easy to use a new technology.	0.76	0.79
PK	3	I can adapt my teaching to the current knowledge level of my students.	0.62	0.49
CK	3	I have sufficient subject matter knowledge in biology.	0.69	0.73
TCK	3	I can use software specifically designed for teaching biology (e.g., interactive whiteboard tools, glossaries, databases, e-learning platforms).	0.75	0.78
PCK	3	I can support my students in various ways to understand the content of biology.	0.82	0.75
TPK	3	I can adapt the use of different technologies to various teaching activities.	0.84	0.82
TPACK	3	I can plan activities in class so that students can construct different representations of the lesson content in biology, using exactly the digital media that are best suited to this.	0.84	0.76

Due to several missing values in the study sample, multiple imputation (see Section 4.4) was performed for both test instruments (TPB scales and the TPACK short scale). To evaluate the internal consistency of the scales before and after multiple imputation of missing values, Cronbach's alpha (α) coefficients were calculated again for both pretest and posttest responses. The alpha values were higher before imputation across most constructs. This is a common observation, as reliability values often decline slightly after multiple imputation due to reductions in the variance and covariance among scale items (Graham, 2009). Specifically, the subjective norm scale (4 items) showed moderate reliability before imputation (α = 0.72 pretest; α = 0.74 posttest; see Table 1). All main analyses were conducted using the imputed dataset to preserve statistical power and minimize bias due to missing data. Given the overall pattern of reliability and theoretical grounding of the constructs, the retained scales were considered suitable for further analysis.

### 4.6 Statistical analysis

Statistical analyses were conducted using Jamovi (Version 2.6.44; The Jamovi Project, 2022; Revelle, 2019), primarily in RStudio (Version 4.5; R Core Team, 2021). Missing values comprised 10.23% of the data. They were handled via multiple imputation using the R package mice (van Buuren and Groothuis-Oudshoorn, 2011). The imputation model included all demographic variables and relevant scale scores as predictors. A total of 10 imputed datasets were generated using predictive mean matching (PMM), with a fixed random seed for reproducibility. The imputed datasets were used for all subsequent correlation and regression analyses. To address RQ, whether participation in the digital training course led to changes in pre-service teachers' TPACK and behavioral intentions, paired-sample *t*-tests were conducted to compare pretest and posttest scale means. The analyses were run separately on each of the 10 imputed datasets, and the test statistics were pooled using Rubin's Rules. The pooling procedure was performed using R, in line with the recommendations from von Hippel (2020).

To account for the missing data, paired sample *t*-tests were conducted across each of the 10 imputed datasets. Since each dataset represented a slightly different plausible version of the original data, the results from the 10 analyses were subsequently combined using Rubin's rules (Rubin, 1987). This pooling procedure considers two sources of uncertainty: the variability of the *t*-test results within each dataset (within-imputation variance) and the differences in the results across the imputed datasets (between-imputation variance). For each variable pair (e.g., pre–post), the mean difference and its standard error were calculated separately for each dataset. The estimates were then pooled using an R function that combined the average effect size and adjusted the overall standard error to reflect both within- and between-imputation variability. Degrees of freedom were computed accordingly, allowing valid *t*- and *p*-values to be derived. This approach should account for the uncertainty introduced by the missing data and the imputation process.

Cohen's *d* values were calculated using the R package effectsize (Torchiano, 2016). According to Cohen (1988), a *d*-value of 0.2 describes a weak effect; a value of 0.5 indicates a medium effect, and a value of 0.8 denotes a strong effect regarding the intervention. The calculations were carried out in RStudio using the imputed datasets. To address RQ2 and RQ3, which focused on identifying the predictors of post-intervention results concerning behavioral intention and self-efficacy, two path models were specified and estimated using the R package lavaan (Rosseel, 2012). Each model used the posttest value of a target construct as the dependent variable and included multiple theoretically relevant predictors based on the Theory of Planned Behavior (Ajzen, 1991) and TPACK theory (Mishra and Koehler, 2006). We estimated two models: Model A: Behavioral Intention as a function of attitude, subjective norm, TPACK and self-efficacy; Model B: Self-efficacy as a function of attitude, subjective norm, TPACK and intention. The outcome and predictor sub-scales were computed as row means from their respective items for each imputed dataset. Each model was estimated separately from the 10 imputed datasets using robust maximum likelihood estimation (MLR). The standardized path coefficients were then pooled using Rubin's Rules, implemented via the dplyr and mitools packages for R. Standardized regression coefficients (β), standard errors, significance levels, and explained variances (*R*^2^) are reported for each model. Models were just-identified and thus showed perfect fit by definition. Correlations and descriptive statistics were calculated using the psych package for R (Revelle, 2019). The combination of imputation, pre-post comparison, and path model provides a robust analysis of how the training intervention affected attitude, self-efficacy, and behavioral intentions, and how these outcomes are influenced by key psychological and pedagogical factors.

## 5 Results

### 5.1 Preliminary results

Given the high correlations between the individual subscales of the TPACK framework (see Table 2), the construct was treated as a unidimensional measure in subsequent analyses. For example, the technological pedagogical knowledge scale (TPK) showed strong correlations with both pedagogical knowledge (PK; *r* = 0.65, *p* < 0.001) and the overall TPACK score (*r* = 0.76, *p* < 0.001). Similarly, there were significant correlations between TPACK and all other sub-dimensions, including technological knowledge (TK; *r* = 0.55, *p* < 0.001), content knowledge (CK; *r* = 0.52, *p* < 0.001), and pedagogical content knowledge (PCK; *r* = 0.44, *p* < 0.001). These results indicated a high degree of convergence among the sub-dimensions, suggesting that they collectively reflect a coherent underlying construct.

Additionally, a very strong reliability was observed throughout all subscales (α = 0.92), indicating that the overall scale considers an underlying construct. This analytical approach is in line with previous research by Zinn et al. (2022), who used an adapted TPACK scale in a large-scale study with pre-service teachers and treated the instrument as a compact indicator for digitalization-related professional competence. Their results demonstrated strong internal consistencies across subscales (α = 0.81 to .92) and substantial intercorrelations, supporting the interpretation of the construct as a unified dimension of teacher knowledge in digital contexts. Similar arguments for this approach are also made in Brändle et al. (manuscript in preparation), who argue for the pragmatic and empirical justification of using aggregated TPACK measures in higher education research.

For the purpose of parsimony and to reduce model complexity, only the overall TPACK score was included in the path models rather than analyzing the individual sub-components separately (Table 3).

**Table 3:** Means, standard deviations, and correlations between all TPACK facets for posttest-measurements.

**Scale**	***M***	***SD***	**1**	**2**	**3**	**4**	**5**	**6**
1. TK	2.69	0.81	–					
2. PK	2.88	0.51	0.39^***^	–				
3. CK	2.68	0.66	0.40^***^	0.49^***^	–			
4. TCK	2.61	0.80	0.56^***^	0.43^***^	0.38^***^	–		
5. TPK	2.79	0.69	0.54^***^	0.65^***^	0.55^***^	0.57^***^	–	
6. PCK	2.89	0.65	0.32^***^	0.53^***^	0.58^***^	0.37^***^	0.54^***^	–
7. TPACK	2.67	0.69	0.54^***^	0.60^***^	0.44^***^	0.61^***^	0.73^***^	0.49^***^

CK, Content knowledge; PK, Pedagogical knowledge; TK, Technological knowledge; PCK, Pedagogical content knowledge; TCK, Technological content knowledge; TPK, Technological pedagogical knowledge. ^***^p < 0.001.

### 5.2 RQ1: paired *t*-tests

To address RQ1 (“Does participation in the digital training course lead to changes in pre-service biology teachers' TPACK and behavioral intention to use digital incremental scaffolds?”), we conducted paired-samples *t*-tests comparing pre- and post-training scores. The Shapiro-Wilk test was applied to all variables to test the normality of the distributions. Test statistics ranged from *W* = 0.97 to *W* = 0.98. For all other variables, the assumption of normality was not violated (*p* > 0.05). Highly significant increases were recorded between pre- and post-test scores in all investigated items of the theory of planned behavior scales and the TPACK short scale. A medium to high effect size was recorded for the attitude and self-efficacy to implement the methods of digital incremental scaffolds after the intervention, and for overall TPACK and the behavioral intention to use the methods in future biology lessons. To evaluate the effects of the training course on motivational and cognitive variables, paired-sample *t*-tests were conducted for each of the five target constructs. Bonferroni correction was applied across the five dependent variables to adjust for multiple comparisons, resulting in a corrected alpha level of α = 0.01. Accordingly, only *p*-values below this threshold were considered statistically significant. The results are summarized in Table 4.

**Table 4:** Paired sample *t*-tests (pre-and posttests) (*N* = 100).

**Scale**	**Pre**		**Post**		***df***	***t*-test**	**Shapiro-Wilk (W)**	**Cohen's *d***
	*M*_Pre_	*SD*_Pre_	*M*_Post_	*SD*_Post_				
SN	2.38	0.75	2.76	0.61	39.0	3.05^*^	0.23	0.55
SE	1.95	0.83	2.92	0.59	98.0	8.89^***^	0.14	1.33
A	2.37	0.53	2.79	0.44	71.0	5.75^***^	0.18	0.86
I	3.01	0.57	3.45	0.54	82.0	6.04^***^	0.06	0.80
TPACK	2.10	0.90	2.67	0.69	318.0	5.61^***^	0.07	0.70

SN, Subjective norm; SE, Self-efficacy; A, Attitude; I, Intention. ^*^p < 0.01, ^***^p < 0.001.

The degrees of freedom (*df* ) reported for the pooled *t*-tests were based on Rubin's rules for combining estimates across multiply imputed datasets. Unlike classical paired-sample *t*-tests, where the *df* correspond directly to the sample size (e.g., n – 1), pooled estimates account for two sources of uncertainty: the within-imputation variance (U) and the between-imputation variance (B). When the imputations yield highly consistent results (i.e., minimal between-imputation variance), the resulting pooled degrees of freedom can exceed the original sample size. This reflects increased statistical precision due to stable estimates across imputations. In contrast, when there is notable variation across imputations (uncertainty introduced by missing data), Rubin's method appropriately reduces the degrees of freedom. The lower *df* in such cases signals that the model accounts for this uncertainty, providing a more conservative and honest estimation of statistical significance.

There were significant improvements in all constructs after applying the Bonferroni correction. The increase in subjective norm remained significant (*p* < 0.001). Participants' subjective norm significantly increased from pretest (*M*_*SN*_ = 2.38, *SD*_SN_ = 0.75) to posttest (*M*_SN_ = 2.76, *SD*_SN_ = 0.61), *t*_(39)_ = 3.05, *p* < 0.01, indicating a small to medium effect (*d* = 0.55). In contrast, self-efficacy increased significantly from *M*_SE_ = 1.95 (*SD*_SE_ = 0.83) at pretest to *M*_*SE*_ = 2.92 (*SD*_SE_ = 0.59) at posttest, *t*_(98)_ = 8.89, *p* < 0.001. This change indicated a large effect (d = 1.33). Similar patterns were observed for attitude, which increased from *M*_A_ = 2.37 (*SD*_A_ = 0.53) to *M*_A_ = 2.79 (*SD*_A_ = 0.44), *t*_(71)_ = 5.75, *p* < 0.001, *d* = 0.86. Participants also reported a significant increase in behavioral intention, from *M*_I_ = 3.01 (*SD*_I_ = 0.57) to *M*_I_ = 3.45 (*SD*_I_ = 0.54), *t*_(82)_ = 5.75, *p* < 0.001, indicating a medium to large effect (*d* = 0.80). Lastly, TPACK scores improved from *M*_TPACK_ = 2.10 (*SD*_TPACK_ = 0.90) to *M*_TPACK_ = 2.67 (*SD*_TPACK_ = 0.69), *t*_(318)_ = 5.61, *p* < 0.001, corresponding to a medium effect (*d* = 0.70).

In summary, there were significant improvements in all five constructs following the intervention. The strongest changes occurred in self-efficacy, followed by attitude, intention, and TPACK. Subjective norms also improved significantly, although with a smaller effect size. These results suggest that the training was effective in strengthening participants' confidence, motivation, and technological-pedagogical knowledge related to digital scaffolding strategies.

### 5.3 RQ2: regression model for intention

RQ2 (“Which factors predict pre-service biology teachers' behavioral intention to use digital incremental scaffolds?”) was examined using multiple regression analysis with attitude, subjective norm, and self-efficacy as predictors. Pearson correlation coefficients were calculated to examine the relationships between the central constructs after the training intervention (see Table 5). The findings revealed several theoretically plausible and statistically significant associations, particularly between motivational beliefs and behavioral intention. The strongest positive correlation was between attitude and self-efficacy (*r* = 0.64, *p* < 0.001), indicating that participants who felt more confident in implementing digital scaffolding strategies were also more likely to evaluate these strategies positively. This suggests a meaningful interplay between competence beliefs and evaluative judgments–a pattern consistent with findings from educational psychology, where increased self-efficacy is known to foster more favorable attitudes toward pedagogical innovation (Bandura, 1997; Lazarides and Warner, 2020).

**Table 5:** Means, standard deviations, and correlations between variables (*N* = 100).

**Scale**	***M***	***SD***	**1**	**2**	**3**	**4**
1. Subjective norm	2.76	0.61	–			
2. Self-efficacy	2.92	0.59	0.13	–		
3. Attitude	2.79	0.44	0.29^**^	0.64^***^	–	
4. Behavioral intention	3.45	0.54	0.31^**^	0.31^**^	0.63^***^	–
5. TPACK	2.67	0.69	0.12	0.55^***^	0.39^***^	0.14

^**^p < 0.01; ^***^p < 0.001.

**Figure 5:**
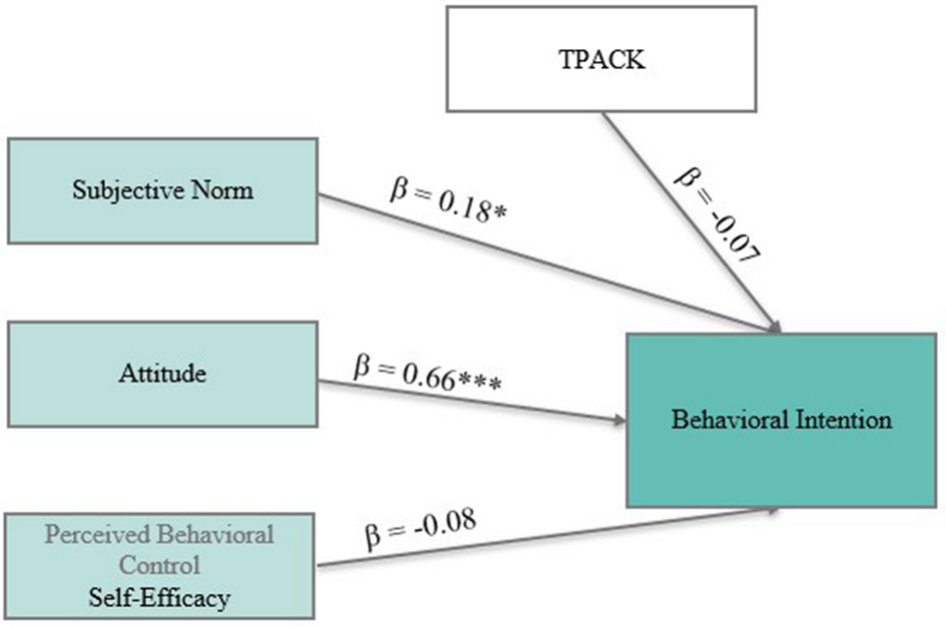
Model A: A standardized path model predicting pre-service biology teachers' behavioral intention to implement digital incremental scaffolds. Significant paths are marked with asterisks (^*^p < 0.05; ***p < 0.001).

Model A (Figure 5) focused on behavioral intention as the response variable. The model was just identified and accounted for 44.3% of the variance in intention (*R*^2^ = 0.443). These ideal values are a result of the models being just-identified (saturated), meaning that all possible direct paths between the predictor variables were specified. Consequently, there are no degrees of freedom left, and the fit indices cannot provide information about the adequacy or parsimony of the model. While saturated models always show perfect fit, this does not necessarily indicate a theoretically optimal or parsimonious model. Therefore, the interpretation must rely on the significance and strength of individual path coefficients (standardized estimates) as well as the explained variance (*R*^2^ values) of the response variables. Among the predictor variables, attitude toward digital scaffolds emerged as the strongest and most significant predictor (β = 0.66, *p* < 0.001). This suggests that participants who viewed digital scaffolds more favorably were more likely to intend to implement them in their future teaching. In addition, subjective norm was a significant predictor (β = 0.18, *p* < 0.05), indicating that social expectations (e.g., from seminar instructors or colleagues) also contributed positively to the formation of intention. In contrast, (perceived behavioral control) self-efficacy (β = −0.08, *p* = 0.54) and TPACK (β = −0.07, *p* = 0.50) did not significantly predict intention.

Behavioral intention—the central outcome in the Theory of Planned Behavior—was significantly associated with attitude (*r* = 0.64, *p* < 0.001), subjective norm (*r* = 0.31, *p* < 0.01), and self-efficacy (*r* = 0.31, *p* < 0.01). These findings are closely aligned with the assumptions of the TPB (Ajzen, 1991), which posits that intention is driven by personal beliefs (attitude), social influence (subjective norm), and perceived behavioral control (PBC). Furthermore, self-efficacy was moderately correlated with TPACK (*r* = 0.55, *p* < 0.001), indicating that participants who felt more confident in their ability to implement scaffolds also reported greater knowledge of pedagogical-technological integration. This supports the notion that domain-specific competence, such as the ability to link technology, pedagogy, and content, can strengthen motivational beliefs (Tondeur et al., 2017). In contrast, the correlation between TPACK and behavioral intention was weak and non-significant (*r* = 0.14, n.s.), suggesting that knowledge or competence alone may not be sufficient to motivate intended implementation. This is consistent with previous research (e.g., Habibi et al., 2022) that suggests that belief-related constructs—especially attitudes—as more direct predictors of intention than knowledge-based constructs.

### 5.4 RQ3: correlation between subscales and regression models for self-efficacy

In order to answer RQ3 (“How do motivational beliefs and TPACK interact in predicting pre-service biology teachers' self-efficacy?”), we estimated a regression model with TPACK and the TPB components as independent variables and self-efficacy as the outcome. Taken together, the correlation patterns (see Table 5) support core elements of the TPB: attitude, subjective norm, and self-efficacy are key psychological predictors of behavioral intention. The data also point to a potentially mediating or supporting role of TPACK, which may contribute more indirectly by enhancing self-efficacy or shaping attitudes, rather than functioning as a direct driver of intention. While the correlation analysis sheds light on bivariate associations among the constructs, regression modeling allows for a deeper understanding of their predictive relationships.

**Figure 6:**
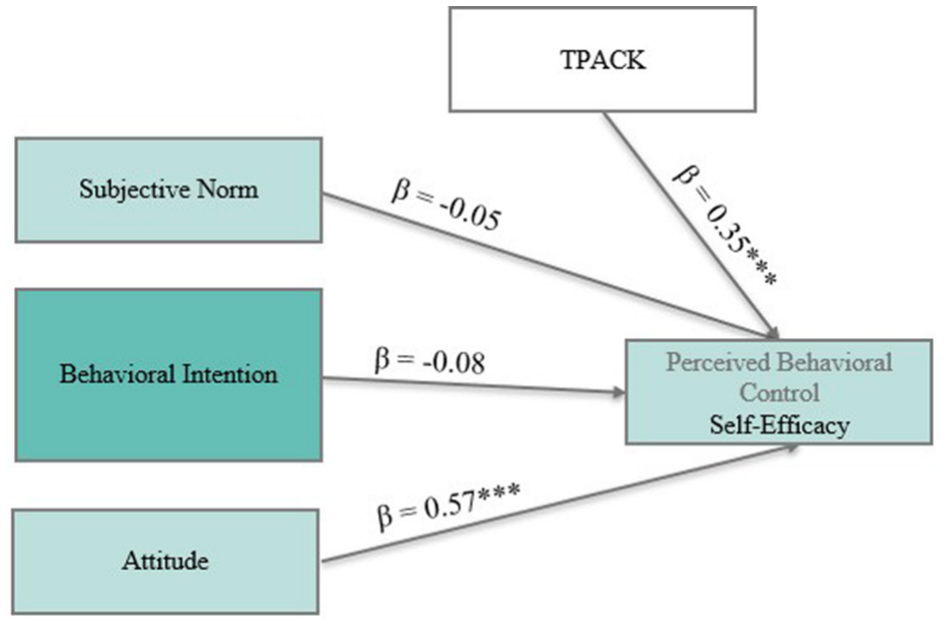
Model B: A standardized path model predicting pre-service biology teachers' self-efficacy to implement digital incremental scaffolds. Significant paths are marked with asterisks (***p < 0.001).

Model B (see Figure 6) examined self-efficacy as the outcome variable. These findings indicate that participants who felt positively about digital scaffolding strategies and perceived themselves as more competent in integrating technology and pedagogy also felt more confident in their ability to create such scaffolds. Other predictors, including subjective norm (β = −0.05, *p* = n. s.) and behavioral intention (β = −0.08, *p* = n. s.), were not significantly related to self-efficacy.

Attitude emerged not only as a key predictor of intention but also as a reinforcing factor for self-efficacy, underscoring its central role in the Theory of Planned Behavior. While TPACK does not directly influence intention, it contributes to the development of self-efficacy, suggesting its relevance for building confidence in digital teaching practices. Notably, the absence of a reciprocal relationship between perceived control and intention challenges the assumptions of a fully circular motivational model, emphasizing the need to distinguish between different motivational constructs when designing interventions to support digital innovation in teacher education.

To conclude, the pre-post comparisons revealed statistically significant increases in both behavioral intention [*t*_(71)_ = 6.04, *p* < 0.001, *d* = 0.80] and self-efficacy expectations [*t*_(98)_ = 8.89, *p* < 0.001, *d* = 1.33], indicating that the digital training was effective in enhancing these outcome variables. In line with the Theory of Planned Behavior, the regression model predicting behavioral intention revealed that attitude (β = 0.66, *p* < 0.001) and subjective norm (β = 0.18, *p* < 0.01) were significant predictors, while self-efficacy, controllability, and TPACK did not contribute significantly. Moreover, a separate model predicting self-efficacy revealed that both attitude (β = 0.57, *p* < 0.001) and TPACK (β = 0.35, *p* < 0.001) were significant predictors.

## 6 Discussion

In this digital era, teachers need professional digital competency to foster their learners' use of digital tools (Dolezal et al., 2025). A previous study of American in-service teachers revealed a strong correlation between their initial beliefs and their intention to participate in ongoing professional development (Dunn et al., 2018). Behavioral intention was significantly predicted by perceived behavioral control, subjective norm, and attitude. The analysis identified perceived behavioral control as the most powerful predictor of behavioral intention. Formal experiences for professional development like structured workshops may be necessary to support the development and improvement of (pre-) service-teachers' competence (Makhmetova et al., 2025).

Digital competence is an essential component of the skill set required in the twenty-first century, and it is expected that it will be taught at school. Therefore, universities should focus not only on mediating subject-specific knowledge but also on developing and implementing a holistic and adaptable system that integrates digital competence into the teacher education curriculum (Dolezal et al., 2025).

This discussion is organized in three parts, and is centered on the three central research questions: first, regarding the effectiveness of the training; second, regarding the predictors of behavioral intention and third, predictors of perceived behavioral control (self-efficacy).

### 6.1 Effects of the training intervention (RQ1)

With regard to RQ1 (“Does participation in the digital training course lead to changes in pre-service biology teachers' TPACK and behavioral intention?”), we observed significant increases in attitude, self-efficacy, behavioral intention and TPACK. These results are in line with previous research highlighting the value of self-paced online modules in strengthening motivational beliefs and confidence in the use of technology (e.g., Sadaf et al., 2012; Lohrsträter et al., 2025). In particular, the increase in self-efficacy demonstrates that competence-oriented training formats with practical digital examples can support the development of technology-related teacher self-beliefs, factors that are considered crucial for later implementation in the classroom (Tondeur et al., 2017). Although the effect sizes were in the small-to-moderate range, they are meaningful in light of the relatively short duration of intervention (approximately 3 hours) and the asynchronous online format. These findings demonstrate that even low-threshold digital training formats can produce measurable motivational and cognitive gains when grounded in theoretical models and focused on pedagogical application. This is in line with previous findings from Bwalya et al. (2023). These indicate that acquiring knowledge of TPACK and engaging with technological tools for teaching and learning, such as PhET simulations (Physics Education Technology, Banda and Nzabahimana, 2021), significantly contributes to the growth and development of technological expertise among pre-service biology teachers. Furthermore, experimenting with these technologies greatly enhanced their technical competencies and their confidence in applying them in lessons. These discussions enabled students to clarify common misconceptions about biological concepts and improve their integration of content, technology and pedagogical knowledge. Lastly, providing pre-service teachers with the opportunity to teach a lesson gave them hands-on experience in delivering technology-rich lessons and helped them internalize their TPACK knowledge. Furthermore, the findings suggest that behavioral intention and self-efficacy, although conceptually related, may be driven by different underlying mechanisms. While intention appears to be shaped by motivational variables such as attitude and social expectations, self-efficacy is more closely linked to knowledge and perceived competence. This supports the idea that effective training must not only address knowledge acquisition but also create opportunities for teachers to internalize the value and feasibility of creating a digital scaffolding.

### 6.2 Predictors of behavioral intention (RQ2)

RQ2 asked which belief-related factors predict pre-service teachers' intention to implement digital incremental scaffolds. The regression analysis (Model A) showed that attitude and subjective norm significantly predicted behavioral intention, while perceived behavioral control/self-efficacy was less influential. These findings suggest that pre-service teachers' internal evaluations of usefulness and perceived social expectations are key drivers of implementation intentions. This could suggest that internal evaluations of the usefulness of digital scaffolds (attitude) and perceived social expectations (subjective norm; e.g., from teacher educators or school environments) are key factors shaping pre-service teachers' implementation intentions. These results align with prior findings that emphasize attitude as the strongest driver of intention in the context of educational technology (Teo, 2011) and partially support the TPB assumption that intention is shaped by both personal and social beliefs. In TPB studies, attitude is a strong predictor of the intention to engage in a behavior, and suggested ways to improve behavior often involve describing the positive consequences of a proper attitude. This could encourage pre-service teachers to consider and apply competency-based practices, differentiated instruction, and formative assessments as integral parts of their regular teaching. Interestingly, while subjective norm is often a weaker predictor, the current results highlight its meaningful contribution. Social influences, including the support of mentors, peers, and educational leaders, play a meaningful role in shaping teachers' beliefs and intentions (Mkhomi et al., 2025). Integrating opportunities for collaboration and peer exchange into professional learning environments can strengthen subjective norms and motivate sustained engagement with digital teaching tools. This is in line with the findings of Leiss et al. (2025) who emphasized the relevance of social influences in shaping technology-related beliefs. Therefore, social support should be explicitly integrated into professional learning environments to maximize motivational outcomes.

### 6.3 Predictors of behavioral intention (RQ3)

Turning to RQ3 (“How do motivational beliefs and TPACK interact in predicting self-efficacy?”), the results of regression model B underline the interdependence between TPACK and perceived behavioral control. While TPACK did not significantly predict intention (RQ2), it emerged as a strong predictor of self-efficacy. The results from regression model B underline the interdependence between self-belief and pedagogical-technological knowledge: those who feel confident in their ability to effectively use technology in teaching tend to also report higher self-efficacy in implementing scaffolding strategies. This suggests that the behavioral intention itself may function as a motivational resource that reinforces beliefs about one's teaching ability. Interestingly, while TPACK was not a significant predictor of intention, it was a strong predictor of self-efficacy. This discrepancy suggests a potential distinction between motivational and competence-based constructs within the TPB framework. Intention seems to rely more on attitude and social norms, while self-efficacy is more strongly linked to domain-specific knowledge and perceived readiness. This echoes the findings of Habibi et al. (2022) and Leiss et al. (2025), indicating that the determinants of motivation and competence are not interchangeable and should be treated as distinct constructs in teacher education research.

Recent findings indicate that even brief, low-threshold professional development formats such as asynchronous online modules lasting only 3 h, can produce significant gains in teachers' attitudes, self-efficacy, and behavioral intentions (Darling-Hammond et al., 2017). This underlines the potential of short, practice-oriented training to support the development of digital competence in a resource-efficient and scalable way, particularly in the context of teacher education.

### 6.4 Bridging the intention–behavior gap

Despite an increase in behavioral intention, previous studies have identified a potential gap between what pre-service teachers intend to do and what they actually implement in practice (Hou et al., 2022). Our findings also show [as emphasized by Leiss et al. (2025)] a significant increase in intention.

A study by Aptyka and Großschedl (2022) found that the strongest predictor of pre-service biology teachers' intention to teach evolution was their attitude toward teaching evolution. Teachers who viewed evolution education positively were more likely to intend to teach it. Subjective norms, or perceived expectations from others, also play a role in influencing intention and shaping attitudes. Perceived behavioral control, or confidence in the ability to teach, supports intention, especially when paired with solid knowledge concerning evolution. Additionally, the perceived usefulness of teaching evolution positively affects attitude. Overall, these factors together explained over 65% of the variance in pre-service teachers' intention to teach evolution. However, since we did not assess actual behavior, we cannot draw firm conclusions about whether this intention will be translated into practice. Future studies should therefore include behavioral follow-ups, such as classroom observations or digital lesson plans, to examine the long-term application of digital competency.

In contrast, a study by Bai et al. (2023) focused on in-service teachers' intention to use technology. Here, TPACK indirectly influenced behavioral intention through self-efficacy and attitude toward use. Notably, self-efficacy had a direct effect on intention as well as an indirect effect via shaping a more positive attitude.

Taken together, these studies demonstrate that confidence, knowledge, and perceived value significantly influence teachers' intentions to adopt specific educational practices, whether teaching evolution or integrating technology. In particular, Bai et al. (2023) found that, for in-service teachers, the strongest indirect effect on behavioral intention was observed in the pathway from TPACK to technological self-efficacy and subsequently to behavioral intention. This means that teachers' professional knowledge about technology (TPACK) indirectly increased their willingness to use digital tools in the classroom by first strengthening their confidence in using technology (self-efficacy). In our study, however, the most pronounced effect followed a slightly different pathway: from TPACK to technological self-efficacy, then to attitude, and finally to behavioral intention. This indicates that teachers' attitudes serve as an important mediating factor. In other words, enhancing technological self-efficacy alone may not be sufficient—fostering positive attitudes toward technology appears to be a critical step in encouraging teachers to integrate digital tools into their teaching practice.

## 7 Limitations and future implications

Several limitations should be acknowledged in light of recent findings from research on intervention studies in teacher education (see König et al., 2025). The sample consisted exclusively of pre-service biology teachers, a factor that may limit the generalizability of the findings to other subject areas. Different disciplines may foster distinct attitudes toward digital teaching tools, and the effectiveness of the training format could vary depending on subject-specific demands. First, intervention research in the field often stresses effectiveness testing over theory development, resulting in limited empirical validation of theoretical frameworks (e.g., TPB or TPACK). This reflects a broader trend in recent reviews that have criticized the insufficient integration and operationalization of theoretical constructs across intervention studies (König et al., 2025). The present study employed established models. Controllability as a subdimension of perceived behavioral control, for example, was neglected in this study. In contrast to in-service teachers, pre-service students are less embedded in professional social structures and may experience fewer concrete opportunities for perceived behavioral control, particularly in digital learning environments. For example, items referencing adherence to expectations from superiors (e.g., seminar instructors or school administrators) may not align well with the lived experience of students and may therefore have limited discriminative power (see the item “I follow the convictions of my superiors (e.g., seminar instructors, school principals) in making digital incremental scaffolds a part of my teaching.”). Consequently, the subscale of controllability may be disregarded from further serious analysis.

Moreover, the use of a single-item or overall score for complex constructs such as TPACK may obscure nuanced effects of its sub-dimensions, though similar structures have been explored in Zinn et al. (2022). There, digitalization-related professional competencies were operationalized via an aggregated TPACK score. While such measures are pragmatic and suitable for large-scale assessment contexts, they may mask differentiated influences of individual components, e.g. the distinct roles of pedagogical vs. technological knowledge in shaping attitudes or self-efficacy. For instance, it is conceivable that technological knowledge (TK) primarily predicts self-efficacy, while pedagogical content knowledge (PCK) might relate more directly to instructional decision-making. Future studies should thus consider the trade-off between measurement efficiency and construct specificity. In addition, the use of pre-post designs—though common and practical in teacher education contexts—may limit the causal interpretability of results compared to randomized controlled trials. This limitation also applies to the current study that primarily draws on questionnaire-based data.

Another limitation of this study is the considerable dropout from the total number of course participants (*N* = 287) to the final sample of *N* = 100 pre-service teachers, who voluntarily participated in the scientific studies and were included in the analyses. This raises the possibility of self-selection bias, which may restrict the generalizability of the findings. Future research should aim to minimize dropout, for example by integrating data collection more closely with course participation, and to examine potential differences between respondents and non-respondents.

An additional limitation is the single-group pre-post design without a control condition. While this design is suitable for exploring short-term changes in competence and motivation, it does not allow for strong causal conclusions. Improvements observed after the training may also be influenced by external factors such as concurrent coursework or individual maturation. However, since the training was conducted over a very short period of 2–3 weeks, the likelihood that such external influences substantially affected the results—particularly regarding perceived behavioral control—is relatively low. This strengthens the interpretation that the observed changes are closely linked to the training. Nevertheless, future studies should include comparison groups (e.g., waitlist controls or alternative training formats) to further strengthen causal inference.

Another methodological limitation concerns the short time frame of the evaluation. The lack of long-term follow-up assessments restricts conclusions concerning the sustainability of observed changes in beliefs and intentions. Although intention was significantly increased, no behavioral measures were included to assess actual implementation. This limits our ability to draw conclusions about whether motivational changes translate into actual teaching practice. This is particularly relevant in light of recent findings (e.g., Leiss et al., 2025) that emphasize the need for longitudinal designs to capture delayed effects, transfer of theory into practice, or potential fade-out of training gains over time. In addition, our study design did not include behavioral or performance-based outcomes such as scaffold implementation in teaching scenarios or student learning effects. Such triangulation of data would enrich the interpretation of intervention success. Future research should therefore not only adopt longitudinal designs to examine whether the observed increase in behavioral intention translates into actual classroom practice, and also employ mixed-methods approaches to better understand the mechanisms of behavioral change. For instance, qualitative interviews with participants could shed light on which components of the training (e.g., the demonstration of incremental scaffolds in the basic module) are particularly effective in strengthening self-efficacy or shaping positive attitudes. In addition, follow-up studies should observe whether and how pre-service and in-service teachers actually implement incremental scaffolds in their teaching. Where implementation does not occur, systematic analyses of perceived barriers (e.g., limited time, curricular constraints, or insufficient technical infrastructure) would provide important insights into the challenges of transferring digital innovations into everyday practice. Finally, replication studies in other disciplines such as physics, chemistry, or mathematics would help to determine the extent to which the observed effects generalize beyond biology education.

The findings also provide several practical implications for the design of teacher education programs. First, systematically including practical demonstrations of teaching methods in the base module may strengthen pre-service teachers' self-efficacy and should therefore be considered an essential element of teacher education programs. Second, since subjective norms significantly influenced behavioral intention, universities and training providers should integrate structured opportunities for peer collaboration and mentor feedback, for instance through group lesson planning, peer review, or digital communities of practice. Third, the effectiveness of brief, low-threshold interventions in this study suggests that modular online courses of only a few hours can already produce meaningful gains in motivation and competence. Such formats are resource-efficient and can be scaled across different teacher education contexts. Finally, future programs or revised programs on incremental scaffolds could also address common barriers to their implementation (e.g., limited classroom time, lack of technical infrastructure) and provide strategies to overcome them. Together, these implications highlight that effective professional development requires the combination of competence-building, motivational support, and structural integration into teacher training curricula.

## Data Availability

The raw data supporting the conclusion of this article will be made available by the authors, without undue reservation. Requests to access the datasets should be directed to RK, RL or MO (ricarda.lohrstraeter@uni-koeln.de, rebekka.siedler@ph-ludwigsburg.de, margit.offermann@uni-bielefeld.de).
